# Natural regulatory T cells inhibit production of cytotoxic molecules in CD8^+^ T cells during low-level Friend retrovirus infection

**DOI:** 10.1186/1742-4690-10-109

**Published:** 2013-10-24

**Authors:** Gennadiy Zelinskyy, Tanja Werner, Ulf Dittmer

**Affiliations:** 1 Institut für Virologie des Universitätsklinikums Essen, Universität Duisburg-Essen, Hufelandstr. 55, 45122 Essen, Germany

**Keywords:** Friend retrovirus, Treg, Foxp3, Granzyme, CTL

## Abstract

**Background:**

Cytotoxic T cells (CTL) play a central role in the control of viral infections. Their antiviral activity can be mediated by at least two cytotoxic pathways, namely the granule exocytosis pathway, involving perforin and granzymes, and the Fas-FasL pathway. It was shown that the level of Friend retrovirus (FV) replication determines the cytotoxic pathway for the control of viral infection. In low-level infection only the Fas pathway is active, whereas cytotoxic molecules are not produced. In the current study, we elucidate the role of CD4^+^ regulatory T cells (Tregs) in suppressing the exocytosis pathway during an asymptomatic low-level infection.

**Findings:**

We show that even a low-level retrovirus infection induced a strong activation and proliferation of natural Tregs. The expanded Tregs suppressed the proliferation of virus-specific CD8^+^ T cells and the production of cytotoxic molecules by these cells. Not surprisingly, the in vivo killing activity of these CD8^+^ T cells was rather weak. Selective depletion of Foxp3^+^ Tregs resulted in de novo granzyme production and augmented virus-specific in vivo killing, but did not affect the low-level virus replication.

**Conclusions:**

Expanded natural Tregs determined the cytotoxic pathways of virus-specific effector CD8^+^ T cells during the acute phase of retroviral infection.

## Findings

CD8^+^ cytotoxic T cells (CTL) can eliminate target cells by at least two independent cytolytic pathways, the granule exocytosis pathway and the Fas-FasL pathway [1]. Previously, we compared the CTL response against low-and high-level Friend retrovirus (FV) infection of mice [2]. Low-level infection induced moderate activation of CD8^+^ T cells expressing only FasL, whereas high-level infection resulted in extensive expansion of CD8^+^ effector T cells secreting molecules of the exocytosis pathway. In an *in vitro* model of HBV infection [3] it was also described that virus antigen levels were critical for regulating the quality of antigen-specific CD8^+^ T cells. It was concluded that stronger antigen stimulation is needed for the production of granzymes compared to FasL [4]. Along these lines, a certain density of antigen presented on DC was necessary for the differentiation of cytotoxic CD8^+^ T cells [5]. These publications acknowledge the significance of antigen levels for CD8^+^ T cell cytotoxicity, whereas the mechanisms that suppress the production of cytotoxic molecules in low-level virus infections were unknown. In addition, most of our basic concepts on the regulation of acute anti-viral immune responses are derived from model infections with high viral loads (e.g. LCMV), whereas the immune responses to low-level asymptomatic infections are poorly defined. A more detailed understanding of such infection courses is very important especially in chronic viral infections as acute viral loads have been shown to correlate significantly with chronic viral set points and onset of chronic disease in infections like HCV or HIV [6,7]. In HCV infected patients, an acute low-level asymptomatic infection even results significantly more often in chronicity and thus is a negative prognostic factor [7].

**Figure 1:**
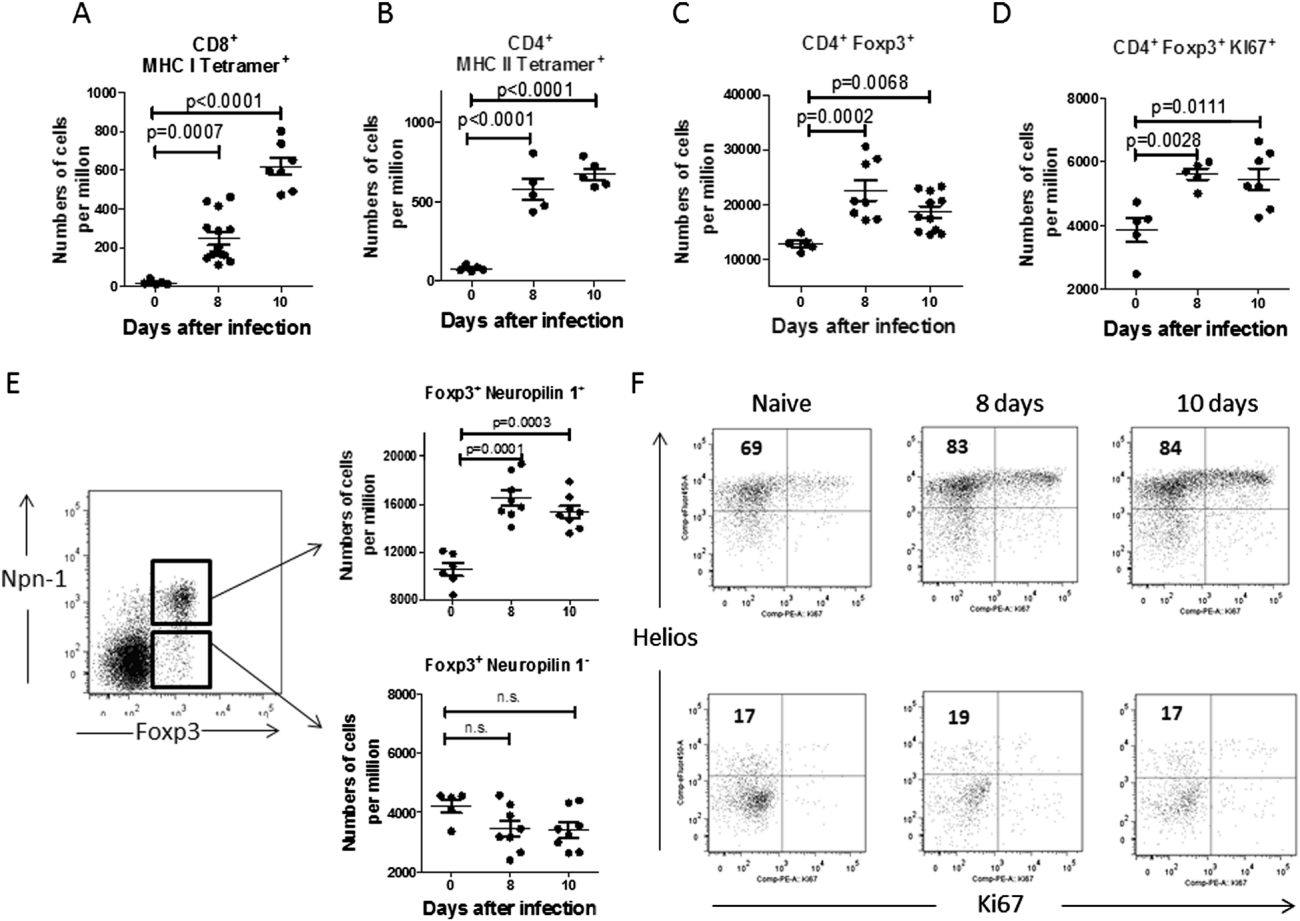
**Expansion of Treg after infection with F-MuLV.** C57BL/6 mice were infected with F-MuLV and sacrificed 8 or 10 days after infection. Then, suspensions of spleen cells were prepared. Flow cytometry was used to quantify the numbers of CD8^+^ T cells specific for the H-2D^b^-restricted F-MuLV glycosylated gag epitope (MHC I Tetramer^+^), and CD4^+^ T cells specific for the A^b^-restricted FV-H19-Env epitope (MHC II Tetramer^+^) in the spleen. **A**. Numbers of CD8^+^ MHC I Tetramer^+^ T cells per one million spleen cells. **B**. Numbers of CD4^+^ MHC II Tetramer^+^ T cells per one million spleen cells. **C**. Numbers of CD4^+^ Foxp3^+^ T cells per one million spleen cells. **D**. Numbers of CD4^+^Foxp3^+^ T cells expressing Ki-67 per one million spleen cells. **E**. A representative dot plot shows the gating-strategy to identify CD4^+^ Foxp3^+^ that were Npn-1^+^ or Npn-1^-^. Upper graph, numbers of CD4^+^Foxp3^+^Npn-1^+^ cells per one million nucleated cells. Lower panel, numbers of CD4^+^Foxp3^+^Npn-1^-^cells per one million nucleated cells. **F**. A representative dot plot shows the expression of Helios and Ki67 on gated CD4^+^Foxp3^+^Npn-1^+^ nTregs (upper panel) and on gated CD4^+^Foxp3^+^Npn-1^-^iTregs (lower panel). The numbers in the upper quadrants represent the percentages of Helios^+^ cells. Data was pooled from three independent experiments with similar results. Differences between naïve mice and mice infected for 10 days with F-MuLV were analyzed by an unpaired t-test. Statistically significant differences between the groups are indicated in the figure.

In our previous studies we showed that during high-level FV infection the population of Foxp3^+^ regulatory T cells (Tregs) follows the expansion of effector CD8^+^ T cells [8] and abrogates the cytotoxicity of these cells during the late phase of acute [9,10] and during chronic infection [11]. Studies on other viral infections also showed that Tregs were involved in the suppression of effector T cell functions [12-15]. Similarly, low-level F-MuLV infection induced the expansion of virus-specific CD8^+^ T cells that recognized the F-MuLV glycosylated gag epitope (Figure 1A). Also CD4^+^ T cells recognizing the FV-H19-Env F-MuLV epitope [16] expanded (Figure 1B). The expansion of virus-specific CD8^+^ and CD4^+^ T cells was detectable at as early as day 8 after infection with F-MuLV and peaked on day 10. Prior to day 8 post infection no effector T cell expansion was detected (data not shown). We wanted to determine whether Tregs also expand during low-level virus infections and if they influence the pathway of CD8^+^ T cell cytotoxicity. To investigate the role of Tregs in C57BL/6 mice with low-level retrovirus infection we performed flow cytometric analysis of CD4^+^ Foxp3^+^ T cells [9] during the acute phase of F-MuLV infection [2]. Virus infection was associated with a moderate but significant enhancement of Treg numbers in the spleen at days 8 and 10 post infection (dpi). At 8 dpi the mean frequency of Tregs was increased by 1.7 fold (Figure 1C). The data document the expansion of Tregs after low-level F-MuLV infection concomitant with the peak CD8^+^ and CD4^+^ T cell response (Figure 1A, B, and C). This finding was surprising as in the case of high-level FV infection the expansion of Tregs was detectable only two days later [9]. By using the proliferation marker Ki-67 [17], we analyzed next whether the expansion of Tregs was a result of the proliferation of CD4^+^Foxp3^+^ cells. In accordance with the expansion of Tregs, significantly more CD4^+^Foxp3^+^ cells expressed Ki-67 at 8 and 10 dpi in comparison to non-infected mice (Figure 1D). Foxp3^+^ Tregs can be divided into thymic-derived “natural” Tregs (nTregs) and Foxp3^-^ conventional T helper cells that, in peripheral tissues, convert into Foxp3^+^ Tregs, so called induced Tregs (iTregs) [18]. We therefore analyzed whether F-MuLV infection resulted in expansion of nTregs or iTregs by using the transmembrane glycoprotein neuropilin-1 (Npn-1) as a marker for nTregs [19,20]. A significantly enhanced frequency of CD4^+^Foxp3^+^Npn-1^+^ (nTreg) was observed at 8 and 10 dpi compared to non-infected mice (Figure 1E upper panel). In contrast, after F-MuLV infection (Figure 1E lower panel), the frequency of CD4^+^Foxp3^+^Npn-1^-^(iTreg) was rather declining instead of increasing. It was recently shown that activated Tregs with enhanced suppressive activity express a member of the Ikaros transcription regulating family, called Helios [21,22]. We analyzed the expression of Helios in combination with the proliferation marker Ki-67 on populations of nTregs (Npn-1^+^) (Figure 1F upper panel) and iTregs (Npn-1^-^) (Figure 1F lower panel). The percentage of activated Helios + cells in the population of nTregs was higher in infected than in naïve mice (83 % to 69 % respectively) (Figure 1F, upper panel), whereas the activation level in the population of iTregs was unchanged after infection with F-MuLV (Figure 1F, lower panel). The combination of activation and proliferation markers allowed us to show that low-level retrovirus infection selectively induced proliferation and activation of nTregs.

**Figure 2:**
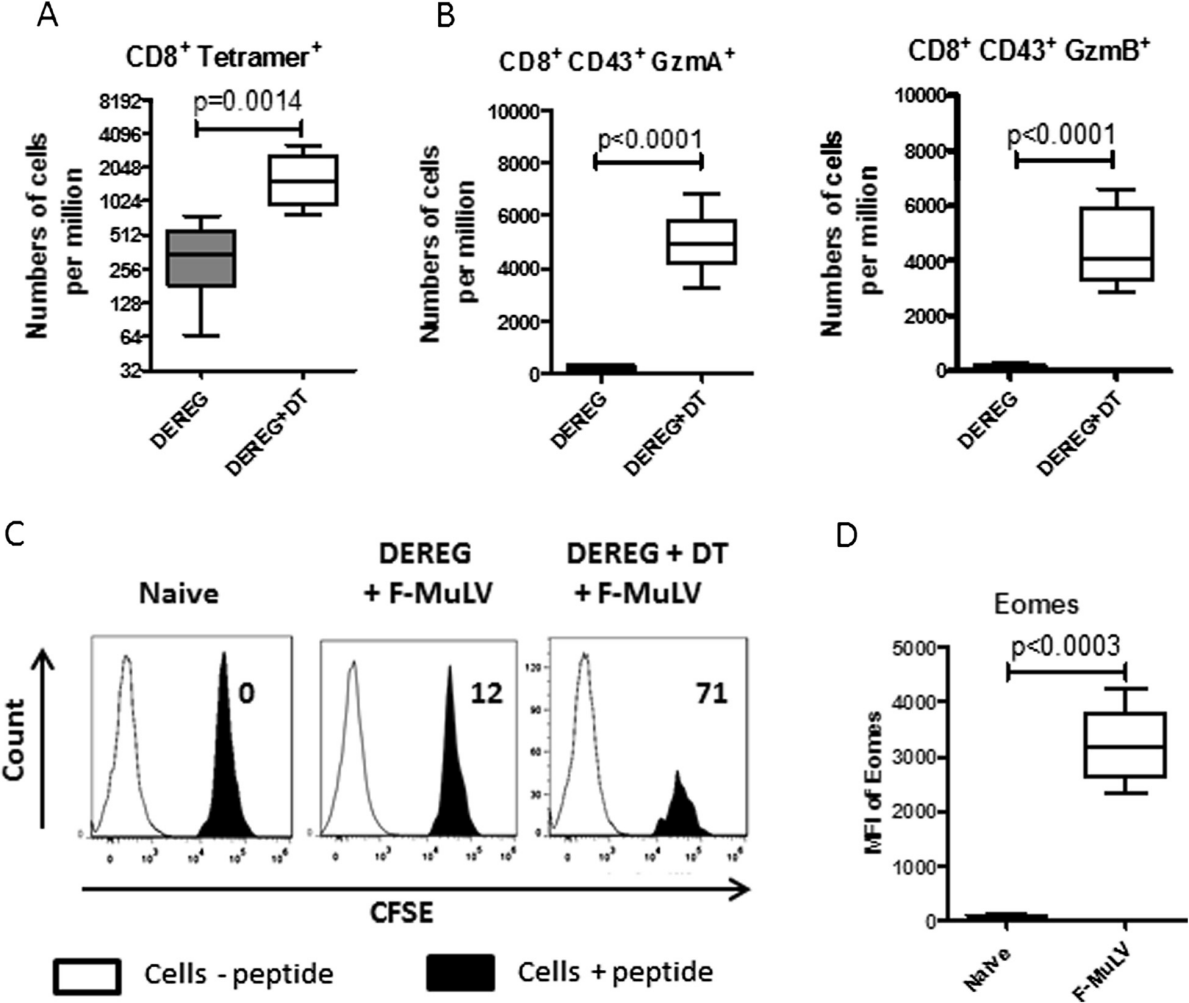
**CD8+ T cell responses in F-MuLV infected Treg-depleted DEREG mice.** F-MuLV-infected DEREG mice were treated with diphtheria toxin (DT) to deplete Foxp3^+^ Tregs during the first 10 days of infection (white columns). Control mice were infected but received PBS instead of DT (black columns). Flow cytometry was used to determine numbers of CD8^+^ T cells reactive with FV-D^b^gagL class I tetramers **(A)**, or numbers of CD43^+^CD8^+^ T cells expressing granzyme A (GzmA) or granzyme B (GzmB) **(B)**. Each column represents mean numbers plus SEM per one million nucleated cells for a group of 5-7 mice. Data was pooled from two independent experiments with similar results. Differences between the groups were analyzed by an unpaired t-test. Statistically significant differences between the groups are indicated in the figure. **C**. Splenocytes from naïve mice were loaded with FV-DbGagL peptides and labelled with CFSE to perform an in vivo CTL assay. As negative control, equal numbers of cells without FV peptide were used from CD45.1 B6 mice. Target cells were injected intravenously into naïve DEREG mice, DEREG mice infected for 10 days or F-MuLV-infected DEREG mice treated with DT. A histogram of CD45.1^+^ CFSE^-^ (transferred cells without peptide) and CFSE^+^ cells (peptide loaded) from the spleen of representative mice for each group (one from a group of 5 animals) is presented. The figure shows the percentage of target cell killing in the spleen. **D**. Flow cytometry was used to determine the mean fluorescence intensity (MFI) of Eomesodermin (Eomes) in CD8^+^ T cells from naïve mice and CD8^+^ T cells reactive with FV-DbgagL class I tetramers from 10 days F-MuLV infected mice. Differences between the groups were analyzed by an unpaired t-test. Statistically significant differences between the groups are indicated in the figure.

To determine the influence of activated nTregs on the population of virus-specific CD8^+^ effector T in vivo we used the DEREG mouse model [23]. Injection of diphtheria toxin (DT) into DEREG mice, which express the GFP and DT receptor under the control of the Foxp3 promoter, depleted more than 95% of the GFP^+^ Tregs in lymphatic organs (data not shown [9,23,24]). Only Tregs but no other cell population was affected by this treatment [24]. During acute F-MuLV infection, activation and expansion of FV-specific CD8^+^ T cells was observed (Figure 2A, [2]). Depletion of Tregs resulted in a highly significant expansion of virus-specific CD8^+^ T cells (Figure 2A), with a 5-times higher mean frequency of tetramer^+^CD8^+^ T cells recognizing the H-2Db-restricted F-MuLV glycosylated gag epitope. However, the most important question was whether or not nTregs would also influence the pathway of CD8^+^ T cell cytotoxicity. We therefore compared the production of the cytotoxic molecules granzyme A and granzyme B in infected mice depleted for Tregs with that of non-depleted mice [9]. As described previously [2], low-level F-MuLV infection did not induce any expression of granzymes in CD8^+^ T cells (Figure 2B). However, after depletion of Tregs, the CD43^+^ effector CD8^+^ T cells started to produce the cytotoxic molecules granzyme A and B. After DT treatment a mean of about 5,000 CD8^+^ T cells per one million nucleated spleen cells produced granzyme A and a mean of about 4,500 CD8^+^ T cells granzyme B (Figure 2B). To analyze if induction of granzyme production correlated with enhanced CD8^+^ T cell cytotoxicity we performed an in vivo CTL assay [9]. We compared the elimination of CFSE-labeled spleen cells loaded with the FV-DbGagL epitope peptide in F-MuLV infected versus F-MuLV infected and DT treated DEREG mice. In F-MuLV infected mice, moderate killing that reached only up to 12% of the target cells was found (Figure 2C). In contrast, CD8^+^ T cells that expanded in the absence of Tregs were highly cytotoxic and eliminated about 70% of the peptide loaded target cells. These findings correlate with our results on the different cytotoxic pathways that are utilized for killing target cells in these two different groups of mice. Whereas only low-level killing by the Fas-FasL pathway was induced by F-MuLV infection [2], more efficient killing by cytotoxic molecules was observed in infected, Treg-depleted mice (Figure 2C). The production of cytotoxic molecules was described to be regulated by the transcription factor Eomesodemin (Eomes) [25]. We therefore compared the expression of Eomes in CD8+ T cells from naïve mice versus virus-specific CD8^+^ T cells from F-MuLV infected mice (Figure 2D). Interestingly, Eomes was highly expressed in tetramer^+^CD8^+^ T cells from infected mice but not detectable in CD8^+^ T cells from naïve mice. The data suggest that the block of production of granzymes by nTregs during low-level infection occurred after the expression of the transcription factor Eomes. This was supported by the finding that Treg depletion did not influence expression levels of Eomes in virus-specific CD8+ T cells (data not shown).

**Figure 3:**
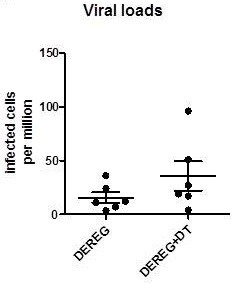
**F-MuLV load in DEREG mice infected with F-MuLV and depleted for Tregs.** F-MuLV-infected DEREG mice were treated with DT to deplete Foxp3^+^ Treg during the first 10 days of infection (white columns). Control DEREG mice were infected but received PBS instead of DT (black columns). Viral loads were measured in the spleen of the 10 days infected mice. Each column represents mean numbers plus SEM per one million nucleated cells. Data was pooled from two independent experiments with similar results. Differences between the groups were analyzed by an unpaired t-test. A statistically significant difference between the groups was not found (P = 0.19).

Our results might lead to the assumption that enhanced functional activation of virus-specific cytotoxic CD8^+^ T cells after Treg depletion results in improved elimination of virus-infected target cells. However, in case of the low-level infection with F-MuLV, which is already well controlled by FasL expressing CD8^+^ T cells [2], the induction of the exocytosis pathway after depletion of Tregs did not result in reduced viral loads compared to infected non-depleted mice (Figure 3). This result indicates that even the limited activation of CD8^+^ T cells by F-MuLV was sufficient for controlling low-level virus replication. In low-level infections, virus-infected cells are rare and it does not seem to be an advantage for the host to have large numbers of highly activated T cells to control virus spread. Instead, the induction of CD8^+^ T cells that produce cytotoxic molecules might enhance the risk of immunopathology during an acute infection. The risk of immunopathology may only be justified if a fast replicating pathogen has to be kept in check, but not during a low-level virus infection. In the later scenario, only moderate CD8^+^ T cell activation with no induction of the potentially dangerous exocytosis pathway is sufficient for virus control. Furthermore, our current data indicate that virus-driven activation and expansion of nTregs is a key factor in down-regulating CD8^+^ T cell activation levels and cytotoxic pathways in low-level infections. Thus, in the case of low-level virus infection, specific CD8^+^ T cells may be functionally suppressed by Tregs, which may result in incomplete elimination of the virus and establishment of chronicity.

## Competing interests

The authors declare that they have no competing interests.

## Authors’ contributions

GZ and UD designed the study. GZ and TW conducted the experiments. GZ performed the data analysis. GZ and UD wrote the paper. All authors read and approved the final manuscript.
